# Choledochoduodenal fistula due to peptic duodenal ulcer diagnosed by X-barium meal study: interest of medical treatment

**DOI:** 10.11604/pamj.2018.29.177.15043

**Published:** 2018-03-26

**Authors:** Mohamed Ali Chaouch, Karim Nacef, Mossab Ghannouchi, Mohamed Ben Khalifa, Asma Chaouch, Molk Abdelkafi, Saida Jerbi, Moez Boudokhane

**Affiliations:** 1 Department of General Surgery, Tahar Sfar Hospital, Mahdia, Tunisia; 2 Department of Radiology, Tahar Sfar Hospital, Mahdia, Tunisia

**Keywords:** Choledochoduodenal fistula, duodenal ulcer, biliary fistula, barium meal

## Abstract

Peptic ulcer complication has decreased over le last years. Spontaneous bilio-digestive fistulas, in the absence of primary biliary disease, remain a very unusual complication of the upper digestive tract. The choledochoduodenal fistula is an extremely rare entity which can be caused by a duodenal peptic ulcer. It appears with the symptoms of peptic ulcer disease. They are diagnosed incidentally on radiological exams. It was suspected after finding pneumobilia on abdominal ultrasound and confirmed by X-barium meals study. The purpose of this observation is to report the case of a patient presenting a choledochoduodenal fistula diagnosed by X-barium meal to underline the importance of this radiological exam to diagnose this disease and to insist on the conservative treatment for choledochoduodenal fistula caused by a duodenal peptic ulcer. The prognosis of patients treated medically is good, although the fistula can remain asymptomatic. Angiocholitis and biliary sequelae remain rare and do not warrant prophylactic surgical treatment.

## Introduction

Internal and spontaneous bilio-digestive fistulas, in the absence of primary biliary disease, remain a very unusual complication of the upper digestive tract. They are largely dominated by cholecystoduodenal fistulas mainly caused by cholelithiasis, which account 90% of bilio-digestive fistulae [1, 2]. A choledochoduodenal fistula (CDF) is an extremely rare entity which can be caused by a duodenal peptic ulcer. Most choledochoduodenal fistulas are diagnosed incidentally by hepato-biliary radiological and endoscopic investigations. The purpose of this observation is to report the case of a patient presenting a CDF diagnosed by X-barium meal to underline the importance of this radiological exam to diagnose this disease and the performance of conservative treatment for CDF caused by a peptic duodenal ulcer.

## Patient and observation

**Figure 1:**
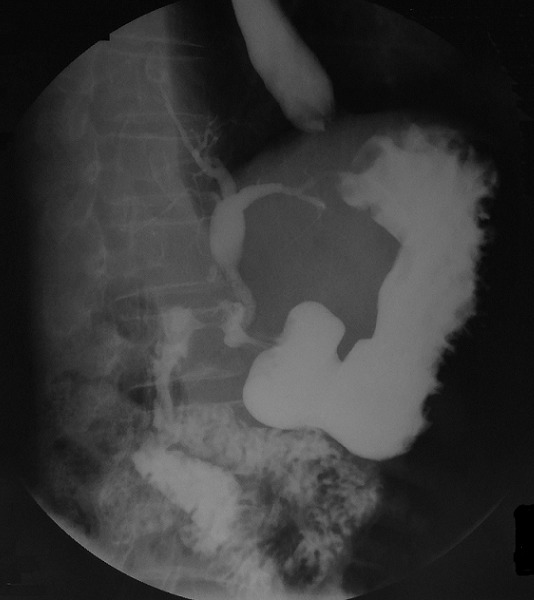
Barium meal study demonstrating deformed duodenal bulb and reflux of contrast into the biliary tract

A 59-year-old man suffering for 9 months of epigastric pain, retro-sternal burns and vomiting. This symptomatology is relieved by proton pump inhibitors. The patient had similar episodes in the past that were treated with Cimetidine for a suspected duodenal ulcer without endoscopic control. On physical examination, the patient was afebrile with mild tenderness in the epigastrium. Biological examinations reveal leukocytosis (12500 cells / mm^3^) with 80% neutrophils without cholestasis and hepatic cytolysis. The abdominal ultrasound showed pneumobilia (the presence of air in the biliary tree) in the absence of dilation of the biliary tract. There were no stones in the gallbladder. Oeso-gastroduodenal endoscopy revealed a duodenal bulb ulcer with erythematous lesions all around. X-barium meal confirmed the existence of a CDF by objectifying a simultaneous opacification of the biliary tract (Figure 1). The diagnosis of CDF due to a bulb peptic ulcer was retained. The decision was to manage the patient conservatively. We opted for an anti-ulcer medical treatment. The evolution was eventful with a clear improvement of the initial symptomatology. An oeso-gastroduodenal endoscopy performed after two months of medical treatment confirm the disappearance of the duodenal ulcer and the choledochoduodenal fistula. The patient remains asymptomatic after 1-year follow-up.

## Discussion

CDF is the abnormal communication between the lower end of the main bile duct and the first duodenal portion [3]. It occurs at the posterior duodenal bulb wall in most cases but occurring in the anterior duodenal bulb wall remain possible. They represent between 3.5% and 8,6% of all biliodigestive fistulas and about 3% are caused by peptic ulcer [4-6]. This affection is uncommon, there were about 80 cases reported in the literature according to publications in Medline database. In 1840, Long was the first to describe a CDF caused by duodenal ulcer [7]. It is underestimated given the large number of asymptomatic CDF. That is why the most cases are incidentally discovered and the preoperative diagnoses still difficult [8]. This affection is more common in men than women, this is due to the frequency of duodenal ulcer disease in men. While fistulas secondary to gallstones are more common in women [9]. Duodenal peptic ulcer communicates usually duodenal bulb with biliary tract into the common bile duct. CDF can be caused by many affections: cholelithiasis, choledocholithiasis, iatrogenic injury, duodenal diverticula, para duodenal abscess, Crohn's disease, liver transplantation, metallic biliary stent placement, cancer of the upper digestive tract and even duodenal tuberculosis [4, 10, 11]. In recent years, the duodenal peptic ulcer is becoming a rare etiology of these CDFs, because of the control and the effectiveness of ulcer drugs than before [4]. It is most often asymptomatic and does not have specific clinical symptoms. Clinical signs are the same of the duodenal peptic ulcer. Angiocholitis, jaundice or anomaly of biological liver exams are rare [1, 10]. For diagnosing CDF, imaging procedures are needed. In some cases, the presence of an internal biliary fistula can be suggested by the findings of pneumobilia, atrophic gallbladder and biliary stones [12]. The accidental discovery of air within the biliary tree on the abdominal X-ray [11] or reflux of contrast in the biliary tree on X-barium meal must suspect the diagnosis of CDF [3]. Oeso-gastroduodenal endoscopy allows the direct visualization of duodenal ulcer, bulbar stenosis and CDF. It's a great way to diagnose in experienced hands. On the other hand, the ERCP with opacification of the fistulous tract is possible but it can be difficult because of the associated ulcer and duodenal deformity but it can be performed with a pediatric endoscope [2, 13]. The abdominal ultrasound can highlight the pneumobilia which presented in our patient and confirm the absence of cholelithiasis that may be the cause of CDF. This pneumobilia is helpful for the diagnosis, but it is present only in 14 to 58% of cases [14]. Moreover, its absence does not eliminate it. The management depends essentially on the etiology of the fistula. Treatment of CDF caused by duodenal peptic ulcer is controversial. Some authors believe that prophylactic surgery is still indicated, others insist that treatment should initially be medical and then surgical in case of failure [3, 4, 14, 15]. However, with the advent of potent anti-ulcer drugs, healing of ulcers has been shown to be associated with healing and closing this type of fistulas like in our case [2]. In addition, patients with high surgical risk treated medically seem to be symptoms relieving in most cases [16]. Some studies suggest that asymptomatic patients probably should not receive treatment, especially surgery. In addition, the risk of angiocholitis or stenosis of the main bile duct is minimal [14, 15]. The endoscopic stent of the bile ducts combined with antiulcer therapy allows the healing of ulcers and fistula. In the past, surgical approaches have been the treatment of choice. Currently, surgery is indicated in patients with a complication of duodenal ulcer: angiocholitis, perforation, bleeding, stenosis or in the absence of improvement by medical treatment [1, 10]. According to the literature, the prognosis of the patients treated medically is good, although the fistula can remain patent [13]. Angiocholitis and biliary sequelae remain rare and do not warrant prophylactic surgical treatment. Then, treatment should focus on the ulcer peptic disease then correction of CDF.

## Conclusion

Spontaneous bilio-digestive fistulas, in the absence of primary biliary disease, remain a very unusual complication of the upper digestive tract. CDF due to the peptic duodenal ulcer is becoming more and rarer. It appears with non-specific symptoms. It is diagnosed generally after radiological or endoscopic investigations. The prognosis of patients treated medically is good. Angiocholitis and biliary sequelae remain rare and do not warrant prophylactic surgical treatment. Then we insist on the conservative treatment for CDF caused by a duodenal peptic ulcer.

## Competing interests

The authors declare no competing interest.
